# Anemia Offers Stronger Protection Than Sickle Cell Trait Against the Erythrocytic Stage of Falciparum Malaria and This Protection Is Reversed by Iron Supplementation

**DOI:** 10.1016/j.ebiom.2016.11.011

**Published:** 2016-11-09

**Authors:** M.M. Goheen, R. Wegmüller, A. Bah, B. Darboe, E. Danso, M. Affara, D. Gardner, J.C. Patel, A.M. Prentice, C. Cerami

**Affiliations:** a Department of Microbiology and Immunology, University of North Carolina School of Medicine, CB# 7435, Chapel Hill, NC 27599-7435, USA; b MRC Unit The Gambia, MRC International Nutrition Group, Keneba, P.O. Box 273, Banjul, Gambia; c University of North Carolina School of Medicine, CB# 9535, Chapel Hill, NC 27599-9535, USA; d Department of Epidemiology, University of North Carolina Gillings School of Global Public Health, CB# 7435, Chapel Hill, NC 27599-7435, USA; e London School of Hygiene & Tropical Medicine, Keppel Street, WC1E 7HT London, UK

**Keywords:** AA, normal β-globin genotype, AC, heterozygous hemoglobin C β-globin genotype, AS, heterozygous sickle-cell trait β-globin genotype, CI, confidence interval, CRP, C reactive protein, G6PD, glucose-6-phosphate dehydrogenase, GPA, glycophorin A, GR, growth rate, Hgb, hemoglobin, IDA, iron deficiency anemia, MCH, mean corpuscular hemoglobin, MCHC, mean corpuscular hemoglobin concentration, MCV, mean corpuscular volume, MFI, mean fluorescent intensity, MPV, mean platelet volume, Pf, *Plasmodium falciparum*, pp, population prevlance, RBC, red blood cell, RDT, rapid diagnostic test, RDW, red cell distribution width, RG, relative growth, SC, heterozygous sickle-cell trait and hemoglobin C β-globin genotype, SD, standard deviation, SI, susceptibility index, SS, homozygous sickle-cell anemia β-globin genotype, sTfR, soluble transferrin receptor, Tf, transferrin, TIBC, total iron binding capacity, Tsat, transferrin saturation, UIBC, unbound iron binding capacity, WBC, white blood cell, Malaria, Iron, Sickle cell trait, Iron supplementation, Hemoglobin, Anemia

## Abstract

**Background:**

Iron deficiency causes long-term adverse consequences for children and is the most common nutritional deficiency worldwide. Observational studies suggest that iron deficiency anemia protects against *Plasmodium falciparum* malaria and several intervention trials have indicated that iron supplementation increases malaria risk through unknown mechanism(s). This poses a major challenge for health policy. We investigated how anemia inhibits blood stage malaria infection and how iron supplementation abrogates this protection.

**Methods:**

This observational cohort study occurred in a malaria-endemic region where sickle-cell trait is also common. We studied fresh RBCs from anemic children (135 children; age 6–24 months; hemoglobin < 11 g/dl) participating in an iron supplementation trial (ISRCTN registry, number ISRCTN07210906) in which they received iron (12 mg/day) as part of a micronutrient powder for 84 days. Children donated RBCs at baseline, Day 49, and Day 84 for use in flow cytometry-based *in vitro* growth and invasion assays with *P. falciparum* laboratory and field strains. *In vitro* parasite growth in subject RBCs was the primary endpoint.

**Findings:**

Anemia substantially reduced the invasion and growth of both laboratory and field strains of *P. falciparum in vitro* (~ 10% growth reduction per standard deviation shift in hemoglobin). The population level impact against erythrocytic stage malaria was 15.9% from anemia compared to 3.5% for sickle-cell trait. Parasite growth was 2.4 fold higher after 49 days of iron supplementation relative to baseline (*p* < 0.001), paralleling increases in erythropoiesis.

**Interpretation:**

These results confirm and quantify a plausible mechanism by which anemia protects African children against *falciparum* malaria, an effect that is substantially greater than the protection offered by sickle-cell trait. Iron supplementation completely reversed the observed protection and hence should be accompanied by malaria prophylaxis. Lower hemoglobin levels typically seen in populations of African descent may reflect past genetic selection by malaria.

**Funding:**

National Institute of Child Health and Development, Bill and Melinda Gates Foundation, UK Medical Research Council (MRC) and Department for International Development (DFID) under the MRC/DFID Concordat.

## Highlights

• *P. falciparum* laboratory and field strains invade and grow less efficiently in RBCs from anemic children.
• Deficits in invasion and growth for erythrocytic stage *P. falciparum* are reversed when RBCs are used from anemic children receiving iron supplementation for 49 and 84 days.
• The population level impact of protection against malaria from anemia was greater than that for sickle-cell trait.

The long-term consequences of anemia are severe, and it is easily treatable. However, concerns remain about the safety of iron supplements, particularly for children in malaria-endemic countries lacking adequate access to health services. We used RBCs from Gambian children before, during, and after 12 weeks of daily iron supplementation for *in vitro P. falciparum* assays. *P. falciparum* invasion and growth was decreased in anemic RBCs and increased after 49 days of iron supplementation relative to baseline (*p* < 0.001), paralleling increases in young RBCs, which the parasite prefers. The parasite growth protection from anemia was substantial, providing greater population level impact than sickle-cell trait.

## 1 Introduction

Malaria and iron deficiency anemia (IDA) impact the same geographic and demographic groups and the pathophysiological relationship between the two is complex. Acute malaria can cause severe anemia due to hemolysis of infected and uninfected RBCs, and chronic or subclinical malaria can induce anemia of inflammation (Clark et al., 2014a). There is clear epidemiological evidence in both children (Gwamaka et al., 2012, Jonker et al., 2012, Nyakeriga et al., 2004) and pregnant women (Kabyemela et al., 2008, Senga et al., 2011) that, once established, IDA is protective against malaria infection. In fact, in pregnant women, iron deficiency has been shown to reduce risk of placental malaria to a greater extent than multiparity (Kabyemela et al., 2008).

Multiple studies have raised concern that iron supplementation in malaria-endemic areas may put people at increased risk of acquiring malaria (Murray et al., 1978, Murray et al., 1975, Oppenheimer et al., 1986, Smith et al., 1989, Veenemans et al., 2011). Most importantly, a large childhood nutritional supplementation study in Zanzibar was halted due to increased morbidity and mortality in children receiving iron (Sazawal et al., 2006). Subsequently, WHO modified its recommendation for universal iron supplementation and now recommends that, in malarious regions, iron supplements be given where malaria management and prevention services are present (Neuberger et al., 2016, World Health Organization, 2016). This has severely disrupted iron supplementation campaigns in malaria endemic areas, despite IDA being the leading cause of years lived with disability among children and adolescents according to the 2013 Global Burden of Disease Study (Global Burden of Disease Pediatrics Collaboration et al., 2016). Reducing the prevalence of anemia is one of the six priorities of the WHO's Comprehensive Implementation Plan on Maternal, Infant, and Young Child Nutrition (World Health Organization, 2014). Further complicating research in this area, it is now difficult to ethically study the safety of iron supplementation in malarious areas. In most developing countries iron supplements cannot be withheld during a study and all children in iron supplementation studies must be provided malaria prevention services and monitored closely for illness. As a result, recent studies evaluating the safety of iron supplementation have done so in the context of providing malaria prevention services and extensive medical care (Mwangi et al., 2015, Zlotkin et al., 2013) – a scenario that would not necessarily exist in reality.

In an effort to assess the magnitude of protection from anemia and the safety of iron supplementation in a malaria endemic area where sickle-cell trait is common, we have systematically characterized *P. falciparum* growth *in vitro* in RBCs from anemic African children before, during, and after 12 weeks of iron supplementation.

## 2 Methods

### 2.1 Subject recruitment, study design, and blood samples for parasite assays

The blood samples for the parasite assays were taken from children enrolled in the control arm of a randomized trial testing the efficacy and safety of a hepcidin-guided screen-and-treat strategy for combatting anemia (see published protocol for full details) (Wegmüller et al., 2016). (Note we also assayed RBCs from children in the other two arms of this trial, but only for observation at baseline, pre-randomization/pre-intervention.) Study participants were recruited from 12 communities in Jarra West (Soma, Karantaba, Kani Kunda, Sankwia, Mansakonko, Pakalinding, Jenoi and Si Kunda) and Kiang East (Toniataba, Jiffin, Kaiaf and Genieri), in the Lower River Region of The Gambia. The study took place from May 2014 through December 2015 in five cohorts. In total 407 healthy young children, aged 6–23 months, were identified during child welfare clinics at the health facilities of Jarra West and Kiang East. After informed consent was obtained, children had to meet the inclusion/exclusion criteria to be enrolled. For inclusion children must have been apparently healthy, 6–23 months old, not severely malnourished (z-scores for Height-for-Age, Weight-for-Age, Weight-for-Height > − 3 SD), 7 g/dl ≤  Hgb < 11 g/dl, free of malaria, resident in the study area, able and willing to comply with the study protocol, have had no congenital disorders or chronic disease, and must not have been taking regular medication nor participating in another study. Sample size was calculated based on the primary endpoint in the parent study (Wegmüller et al., 2016).

**Fig. 1:**
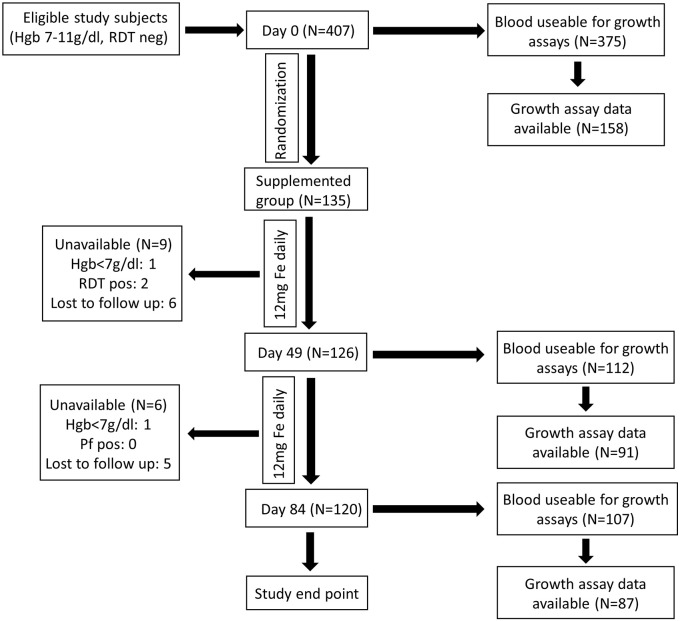
Description of subjects and flow chart of sample collection and assays performed. Blood samples for hematological, biochemical, and parasite growth analyses were drawn at Day 0, as well as Day 49 and Day 84 for those taking iron. A full hematology panel was measured in EDTA-stabilized blood (Medonic M20M GP). We also assayed plasma ferritin, soluble transferrin receptor (sTfR), serum iron, transferrin saturation (TSAT), C-reactive protein (CRP), alpha 1-acid glycoprotein (AGP) (Cobas Integra 400 plus); and hepcidin (Hepcidin-25 (human) EIA Kit (Bachem)). Genotyping for hemoglobinopathies was performed using hemoglobin electrophoresis. Glucose-6-phosphate dehydrogenase (G6PD) enzyme activity was measured by commercial kit (R&D Diagnostics Ltd). For malaria assays, 2.5 ml of venous blood was drawn directly into microvette tubes containing CPDA-1 (Sarstedt, Germany). Unavailable donors include safety exclusion (Hgb < 7 g/dl or positive malaria test, RDT pos) or general loss to follow up (withdrawal and travel). Failure to collect blood from subjects (*e.g.* from phlebotomy failure, subject moved or withdrew, or became significantly ill) was 7.8% (32/407) at Day 0, 17.0% (23/135) at Day 49, and 20.7% (28/135) at Day 84. RBCs from study subjects were evaluated with *in vitro P. falciparum* growth assays (using strain FCR3-FMG) as a proxy measure for malaria susceptibility. In order to standardize the growth assays, control for inter-assay variability and variability between parasite preparations, assays on clinical samples were run in parallel with and reported relative to growth assays done using RBCs from non-anemic donors. Each available blood sample at every time point was subjected to growth assays but not all produced growth data, as some blood was unusable (*e.g.* clotted, hemolysed, contaminated). Further growth data exclusions (*e.g.* parasites died or control blood did not provide a readable output for comparison) do not represent population sampling bias, as subject characteristics are the same between those with and without corresponding growth data (Supplemental Table 2).

As per current WHO recommendations, children in the control arm received 12 mg/d iron as ferrous fumarate, given orally within a micronutrient powder (modified MixMe™ supplied by DSM Nutritional Products). Field workers visited children daily in order to supervise the micronutrient powder administration and check the children's health status. For baseline population characteristics, see Supplemental Table 1. Fresh RBCs were obtained from these anemic (Hgb < 11 g/dl) but otherwise healthy children (6–23 m) living in rural Gambia (Wegmüller et al., 2016). Blood was collected at Days 0 (baseline), 49, and 84 during 12 weeks of iron supplementation (Fig. 1) with the primary objective of evaluating *in vitro P. falciparum* growth characteristics to model malaria susceptibility in anemic subjects before and after iron supplementation. We compared subject characteristics of those whose blood was and was not able to be used for growth rate data to ensure no sampling bias occurred (Supplemental Table 2). For a full description of this embedded observational study, please see the published protocol (Wegmüller et al., 2016).

### 2.2 *P. falciparum* Culture

Parasite lines FCR3-FMG (MR4, MRA-736) and 3D7 (MR4, MRA-102) were routinely cultured in RBCs from healthy donors using standard methods (Clark et al., 2014a). Parasite strains 952, 998, and 1029 were isolated from patients presenting with symptomatic malaria infections at the Jammeh Foundation for Peace hospital in Serekunda and the outpatient clinic at MRC Fajara, both located within the urban/periurban coastal area of The Gambia. Isolates were collected as part of a larger study during the annual malaria transmission seasons (September–January) from 2005 to 2011, as described in (Gomez-Escobar et al., 2010).

### 2.3 2.4 Growth Assay

*In vitro* growth was assessed in fresh, washed RBCs as in (Clark et al., 2014a) for 96 h (performed in triplicate for RBCs from each study participant). RBCs from healthy, iron replete adult donors of normal hemoglobin genotype and G6PD status not undergoing iron supplementation served as controls for inter-assay variability. Growth rates represent final 96 h parasitemia divided by initial 0 h parasitemia (Clark et al., 2014a), analyzed by flow cytometry (see Supplemental methods). Growth rates in subjects' RBCs were normalized to that in control RBCs assayed simultaneously.

### 2.4 RBC Barcoding Invasion Assay

The assay was performed and analyzed as in (Clark et al., 2014b) using two different concentrations of CellTrace Far Red DDAO (Invitrogen Life Technologies/Molecular Probes): 1uM (high) or 0.1uM (low) (see Supplemental methods and Supplemental Fig. 2 for flow cytometry analysis).

### 2.5 Reticulocyte Quantification

Reticulocyte (CD71 +) levels in fresh subject RBCs were assessed using PE-conjugated *anti*-human CD71 antibody (Clone M-A712, BD) and isotype control (Clone G155-178, BD), and analyzed by flow cytometry (see Supplemental methods) for reticulocyte percent relative to non-anemic control.

### 2.6 Statistics

All experiments were done in triplicate. Growth rates, invasion assays, and hematological data were compared by two-tailed Student's *t*-test, one-way ANOVA, and/or 95% CI values using GraphPad Prism 5.

### 2.7 Multivariate Modelling

We employed linear regression to estimate the effect of hematological characteristics on *in vitro* parasite growth rates. First, bivariate associations and their respective 95% CI were calculated between growth rates and hematological and patient characteristics at Day 0. We then used multivariate linear regression. We used directed acyclic graphs to identify potential confounders and controlled for them in our modelling approach (Rothman et al., 2008). An *a priori* alpha of 0.05 was used to determine statistical significance. Analyses were performed using R software (RStudio Version 0.99.902).

### 2.8 Population Level Impact Equation

Using our *in vitro* data on the erythrocytic stage growth of the malaria parasite as a proxy measure for malaria susceptibility, we compared the relative protection offered by sickle-cell trait carriage and anemia using the following formula: pp(RG-1)/RG, where pp is the percentage of the population exposed to the protective factor and RG is the relative *in vitro* parasite growth rate associated with that factor. The RG values for sickle-cell trait and hemoglobin were based on the standardized β coefficients from our multivariate modelling results. In this population of Gambian children, the pp for anemia is 0.75 (derived from 688 children < 3y in the Kiang West Longitudinal Population Study) (Hennig et al., 2015) and the pp of AS is 0.159, (Cox et al., 2008). This calculation does not give an epidemiological measure of disease risk, it is a simple calculation designed to illustrate the relative magnitudes of the impacts of sickle-cell trait and anemia in our study population.

### 2.9 Ethics Approval

The trial from which children were recruited was approved by the MRCG Scientific Coordinating and The Gambia Government/MRC Joint Ethics Committees (SCC 1358) and the UNC IRB (14-1551) which conform to Declaration of Helsinki standards. Parents/guardians were given a full description of the study in their native language and provided written signed consent.

## 3 Results

### 3.1 *P. falciparum* Growth Is Reduced in RBCs from Anemic Children

**Supplemental Fig. 1:**
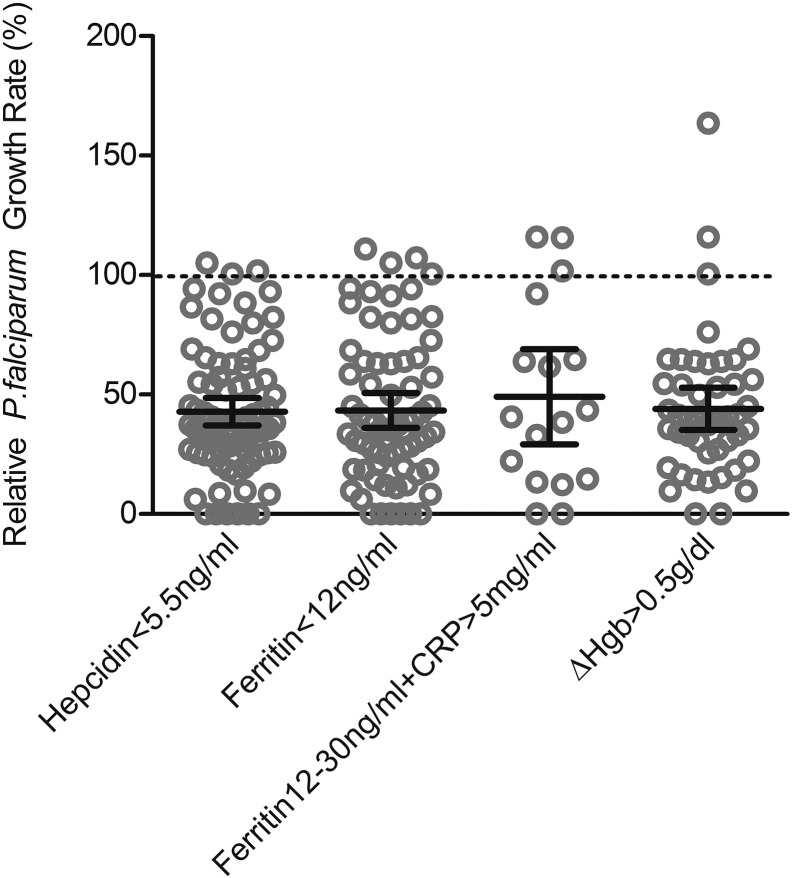
Parasite growth rates in RBCs from children categorized by different definitions of anemia at baseline. In analysis of parasite growth rates in RBCs from children at Day 0, we stratified participants (all anemic) using four different definitions to categorize the severity and type of iron deficiency in the presence or absence of inflammation: those with 1) hepcidin < 5.5 ng/ml (*n* = 82); 2) ferritin < 12 ng/ml (*n* = 69); 3) ferritin12-30 ng/ml with CRP > 5 mg/ml (*n* = 17); 4) hemoglobin increase of > 0.5 g/dl from baseline after 49d or 84d of daily iron supplementation (*n* = 46); definitions 1–4 are not necessarily mutually exclusive. Of note, everyone in our population had a raised serum transferrin receptor (sTfR):log ferritin index > 2 which is highly suggestive of iron deficiency. Growth rate values are presented relative to growth in RBCs from non-anemic donors. Each dot represents the mean result of triplicate growth assays from each donor and the error bars represent 95% CI. Mean growth rate results (with 95%CI) are: hepcidin < 5.5 ng/ml = 42.89% (37.11–48.67%); ferritin < 12 ng/ml = 43.34% (36.01–50.68%); ferritin 12-30 ng/ml with CRP > 5 mg/ml = 49.08% (29.16–69.00%), ΔHgb > 0.5 g/dl = 44.04% (35.14–52.93%). There are no significant differences between the means.

**Fig. 2:**
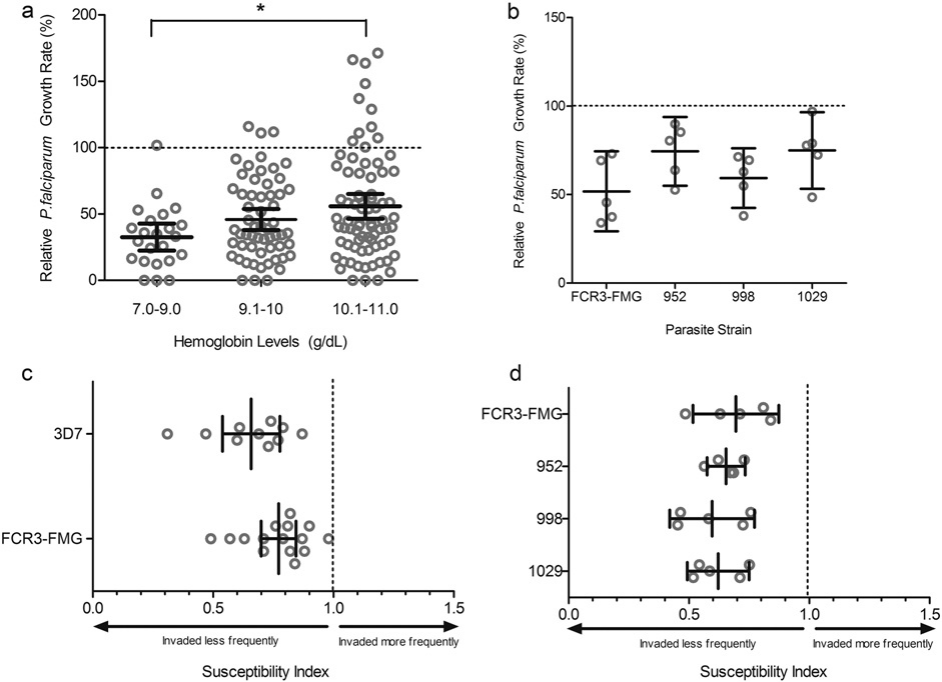
Parasite growth and invasion in RBCs from anemic children (Hgb < 11 g/dl) at baseline. A) *P. falciparum* (strain FCR3-FMG) growth rates are proportional to hemoglobin concentration. Growth assays were performed in RBCs drawn from anemic children at baseline (Day 0) and values are presented relative to growth in RBCs from non-anemic donors. Each dot represents the mean result of triplicate growth assays from each donor and the error bars represent 95% CI. One-way ANOVA indicates the means are significantly different between Days (*p* < 0.05); specifically, post-hoc analysis with Tukey's test indicates significant differences between Hgb levels 7–9 g/dl and 10.1–11 g/dl (*p < 0.05). B) *P. falciparum* clinical isolates from The Gambia exhibit decreased growth in RBCs from anemic children at Day 0. Growth of 3 different clinical strains (952, 998, 1029) was compared to growth of a laboratory strain (FCR3-FMG) in RBCs from five anemic children. Each dot represents the mean result of triplicate growth assays from each donor relative to growth in non-anemic RBCs and error bars represent the 95% CI. The mean relative growth rate in anemic RBCs for each strain is decreased compared to 100% growth in non-anemic RBCs. C) Direct comparison of invasion into RBCs from anemic and non-anemic donors using *P. falciparum* laboratory strains. Invasion experiments for RBCs from all anemic donors (drawn at Day 0) were performed independently and each experiment was performed in triplicate. Data show the mean SI using RBCs from 10 anemic donors for strain 3D7 and 15 for FCR3-FMG. The SI defines the relative susceptibility to invasion of two different types of RBCs. The marker represents the SI point estimate and the bar represents the 95% CI. An SI of 1.0 indicates no difference in parasite invasion of two RBC populations. Both strains 3D7 and FCR3-FMG give SI values significantly decreased from the control value of 1.0. D) Direct comparison of invasion into RBCs from either anemic or non-anemic donors using clinical strains of *P. falciparum*. Invasion experiments for RBCs from all anemic donors (drawn at Day 0) were performed independently and each experiment was performed in triplicate. Data show the mean SI using RBCs from 5 anemic donors for all strains (FCR3-FMG, 952, 998, 1029). The marker represents the SI point estimate and the bar represents the 95% CI. An SI of 1.0 indicates no difference in parasite invasion of two RBC populations.

Evaluating *in vitro* parasite growth in RBCs from anemic children at baseline, we consistently found lower parasite growth rates than in RBCs from iron replete individuals. Furthermore, growth was lower in RBCs from those donors with the lowest hemoglobin concentrations (Hgb 7–9 g/dl = mean relative growth rate (GR) 32.6%; Hgb 8.1–10 g/dl = GR 45.9%; Hgb 10.1–11 = GR 55.9%; *p* < 0.05 by ANOVA) (Fig. 2A). Iron panel data indicated some degree of iron deficiency in most participants (Table 1). However, as the diagnosis of iron deficiency in children with ongoing inflammation is controversial, we grouped subjects using several common definitions of IDA in an attempt to uncover any further differential impacts on malaria susceptibility. We observed decreased parasite growth in all anemic children independent of the type (*e.g.* with inflammation or without) and severity of iron deficiency, with no significant differences between groups (Supplemental Fig. 1).

To further investigate potential confounding effects of inflammation and host genetics on parasite growth, we performed bivariate analysis using *P. falciparum in vitro* growth, hematological, iron, and inflammatory data obtained for subjects prior to iron supplementation to determine which variables influenced parasite growth in anemic children (Table 2). Several key variables commonly assumed to affect anemia and/or blood-stage malaria growth were tested. Hemoglobin genotype influence was evaluated solely based on β-globin sickle-cell trait (AS) mutation *versus* normal β-globin (AA), as other β-globin genotypes (homozygous sickle-cell anemia (SS), hemoglobin C (AC), and a heterozygous combination (SC)) were rare. Hemoglobin concentration, hemoglobin genotype, and mean corpuscular volume (MCV) all significantly influenced parasite growth. G6PD status (normal *versus* deficient) did not significantly affect parasite growth, nor did age, sex, ferritin, hepcidin, or CRP (Table 2). Parasite growth rate decreased 10.7% for every 1 g/dl hemoglobin decrease. Additionally, we found parasite growth rate decreased 1.4% for every 1 fl decrease in MCV and 18.3% in RBCs from children carrying sickle-cell trait. In order to compare the magnitude of these growth rate effects, we standardized the growth rate differences per standard deviation (SD) of each exposure variable, finding 8.6% and 10.8% decreased parasite growth per SD of hemoglobin and MCV, respectively (Table 2). Next, we performed multivariate analysis to determine if the effect of hemoglobin on malaria growth rate was confounded by hemoglobin genotype and *vice versa*. These variables retained significant effects on malaria growth independently of one other, highlighting the independent impact of both microcytic anemia and sickle-cell trait on malaria growth.

### 3.2 The Population Level Impact on Parasite Growth Is Greater from Anemia than Sickle-Cell Trait Genotype

Using our multivariate modelling results, we estimated the population level impact on parasite growth from both sickle-cell trait genotype and anemia in order to assess overall the risk of malaria infection in our study population. Given the prevalence of AS (15.9%) (Cox et al., 2008) and anemia (75%) (Hennig et al., 2015), we thus calculated the population level impact of malaria growth reduction to be 3.5% from sickle-cell trait and 15.9% from anemia in these Gambian children. Note that this underestimates the protection by anemia because it simply compares anemic (defined as Hgb < 11 g/dl, 2 SD below the mean) *versus* non-anemic children. In fact, our population mean Hgb is 3.6 standard deviations below normative data (mean 12 g/dl) from healthy African-American children (Sandoval, 2016); using this comparator the protection offered to the average Gambian child would be a 31% reduction in parasite growth rate (see Table 2).

### 3.3 *P. falciparum* Clinical Isolates Exhibit Decreased Growth in RBCs from Anemic Children

We additionally evaluated the growth of Gambian clinical *P. falciparum* isolates (952, 998, and 1029) to ensure the observed decreased parasite growth in anemic RBCs was not solely a phenomenon of laboratory adaptation. These field isolates assayed in parallel in RBCs from 5 randomly chosen anemic subjects at baseline (with normal hemoglobin genotype and CRP < 5 mg/ml) all exhibited decreased growth compared to RBCs from non-anemic individuals (Fig. 2B). Mean growth rates for all strains were consistently below 100% (FCR3-FMG = 51.88% CI = 29.33–74.43%; 952 = 74.43%, CI = 55.04–93.83%; 998 = 59.34%, CI = 42.51–76.16%; and 1029 = 74.94%, CI = 53.31–96.57%).

### 3.4 RBCs from Anemic Children Are Resistant to Invasion by Laboratory and Field Strains of *P. falciparum*

**Supplemental Fig. 2:**
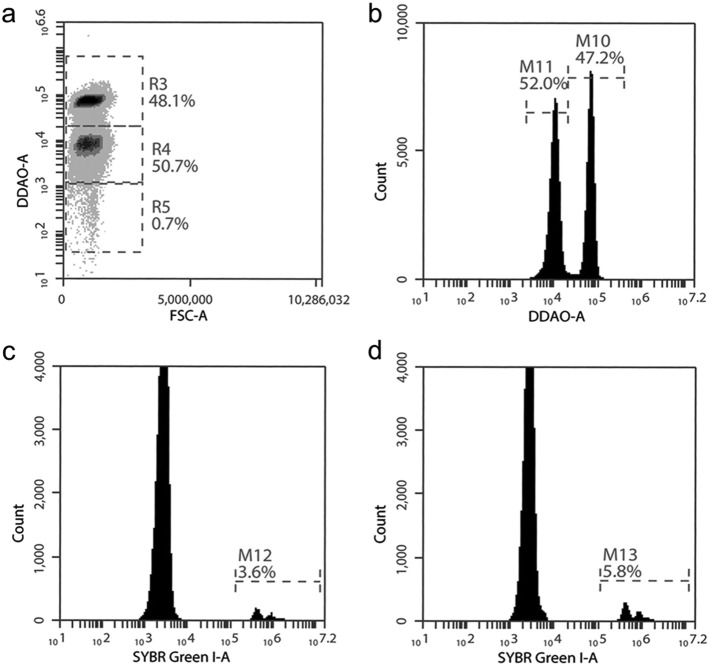
Gating strategy to highlight adaptation of RBC barcoding assay to the field setting using basic two-color flow cytometry. a) RBCs from different blood donors are differentially labelled with CellTrace Far Red DDAO (1 μM (*R*3) or 0.1 μM (R4)) to distinguish between donor populations. Late stage purified parasites grown in unlabeled RBCs (R5) are seeded into the differentially labelled RBCs which have been combined in equal proportion. b) M10 represents the 1 μM Far Red DDAO labelled RBCs from a non-anemic donor and M11 represents the 0.1 μM Far Red DDAO labelled RBCs from an anemic donor. Gating cells on M11 (c) or M10 (d) allows for Sybr Green I DNA dye detection of parasite infected RBCs in the RBCs from anemic donors (M12, c) or from non-anemic donors (M13, d). Parasitemia in each cell population is compared to calculate the invasion SI. The percentages in the flow plots represent the percent of total cells within the indicated gate.

Next, we used a RBC barcoding assay (Clark et al., 2014b) adapted for field use (Supplemental Fig. 2) to directly compare parasite invasion into RBCs from anemic children (*n* = 15 for strain FCR3-FMG and *n* = 10 for strain 3D7) *versus* non-anemic donors. Susceptibility Indices (SI) of RBCs from the anemic donors were significantly decreased using both strains (FCR3-FMG SI = 0.77, CI = 0.70–0.84; 3D7 SI = 0.66, CI = 0.54–0.78) (Fig. 2C). *P. falciparum* clinical isolates from The Gambia (strains 952, 998, and 1029) also exhibited decreased invasion into RBCs from anemic donors (952 SI = 0.65, CI = 0.58–0.73; 998 SI = 0.57, CI = 0.42–0.77; and 1029 SI = 0.62, CI = 0.49–0.75) (Fig. 2D). These assays confirm the clinical relevance of previous *in vitro* work examining laboratory parasite strains and iron deficient RBCs (Clark et al., 2014a).

### 3.5 *P. falciparum* Growth *in vitro* Increases Transiently with Iron Supplementation

**Supplemental Fig. 3:**
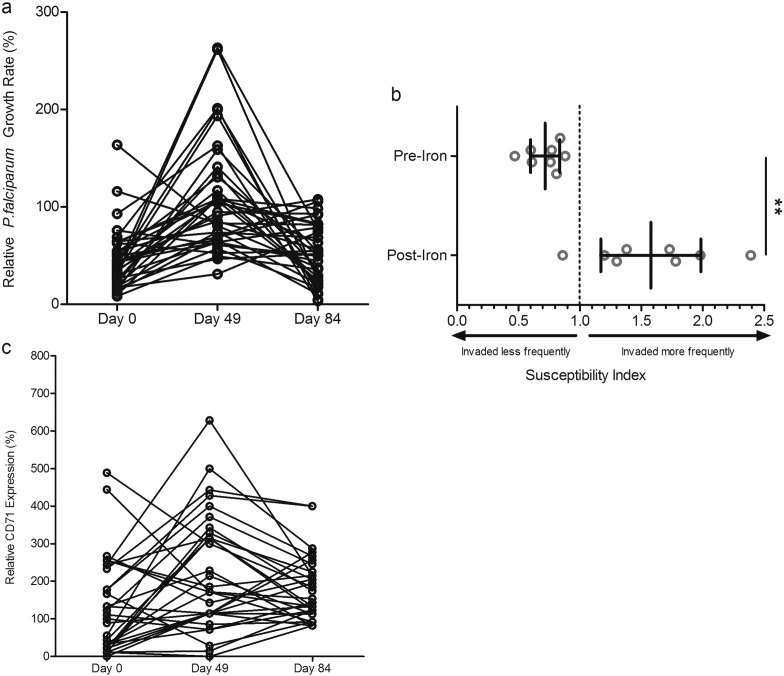
Changes in parasite growth, invasion, and reticulocytosis in RBCs from anemic children before and after daily iron supplementation. a) Levels of parasite growth rates increase over time in anemic children undergoing iron supplementation, as depicted by line graph in order to highlight changes for each individual that had data available at all timepoints (*n* = 35 children with complete repeat growth measures at Day 0, 49, and 84, with 86% having increased growth rate at Day 49) One-way repeated measures ANOVA of growth rate values indicates the means are significantly different between Days (*p* < 0.0001); post-hoc analysis with Tukey's test indicates significant differences between Day 0 and Day 49 means (*p* < 0.001) and Day 49 and Day 84 means (*p* < 0.001), but no significance between Day 0 and Day 84 for those children with repeat measures. b) Direct comparison of invasion into RBCs from non-anemic donors to RBCs from 8 anemic children either before or during 12 mg daily iron supplementation. Each experiment was performed in triplicate for each blood donor. The marker represents the SI point estimate and the bar represents the 95% CI. An SI of 1.0 indicates no difference in parasite invasion of the two RBC populations. Student's *t*-test indicates significant differences between pre- and post-iron SI values (**p < 0.01). c) Line graph of CD71 repeated measures (*n* = 31 children with complete repeat CD71 measures at Day 0, 49, and 84). In 21 of these children, the relative percent CD71 positive cells increased from Day 0 to Day 49. See Fig. 3B for repeated measures ANOVA statistics.

**Fig. 3:**
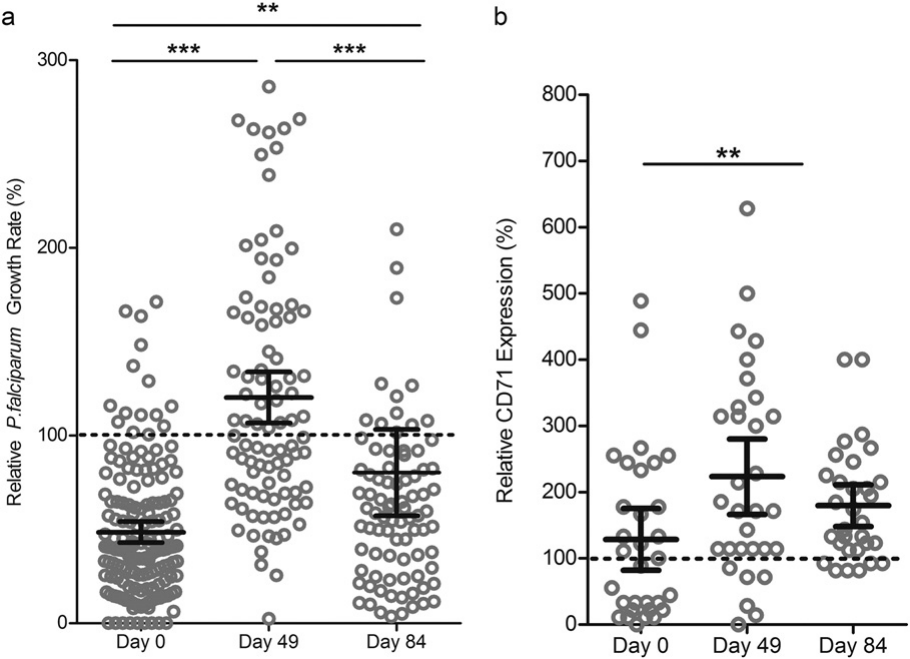
Malaria susceptibility increases transiently during iron supplementation and anemic children receiving iron supplements have increased numbers of young RBCs. A) *P. falciparum in vitro* growth rates in RBCs from anemic children increase over time with iron supplementation (12 mg iron daily for 84 d). Parasite growth assays were conducted in RBCs from children at Day 0, Day 49, and Day 84 using strain FCR3-FMG. Growth rates are reported relative to growth in RBCs from non-anemic donors. Each dot represents the mean of triplicate assays and error bars represent the 95% CI. Differences between growth rates at the different timepoints were significant (*p* < 0.0001 by one-way ANOVA); specifically, post-hoc analysis with Tukey's test indicates significant differences between Day 0 and Day 49 (***p < 0.001) and Day 49 and Day 84 (***p < 0.001), as well as Day 0 and Day 84 (**p < 0.01). *n* = 158 children at Day 0, *n* = 91 children at Day 49, and *n* = 87 children at Day 84. B) Levels of CD71 positive RBCs increase over time in anemic children undergoing iron supplementation. Percent CD71-positive RBCs was measured by flow cytometry analysis of CD71 surface expression. Error bars represent the 95% CI; one-way repeated measures ANOVA indicates the means are significantly different between Days (*p* < 0.01, *n* = 31); post-hoc analysis with Tukey's test indicates significant differences between Day 0 and Day 49 (**p < 0.001) but not between Day 49 and Day 84, nor Day 0 and Day 84.

In order to assess malaria susceptibility following iron supplementation, we investigated *in vitro* parasite growth 49d and 84d after daily iron supplementation compared to baseline. The children were monitored daily for changes in health status and underwent weekly malaria testing. Consistent with the fact that malaria incidence is now low in The Gambia (Mwesigwa et al., 2015), only two malaria cases occurred during our study. Hence, *in vitro* assays offered a way to examine the relationship between growth of malaria parasites in RBCs and changing hematological parameters and capture the window of increased susceptibility. Parasite growth rates in RBCs from study subjects were low on Day 0 (*n* = 158, mean GR 48.51%, CI = 42.88–54.14%), increased markedly by Day 49 (*n* = 91, mean GR 120.3%, CI = 106.6–133.9%), and then by Day 84 decreased back to levels closer to those seen in non-anemic individuals (*n* = 87, mean GR 80.26%, CI = 57.27–103.3%). One-way ANOVA confirmed significant differences in parasite growth rates across Days 0, 49 and 84 (*p* < 0.0001) and post-hoc analysis using Tukey's test indicated significant differences between Days 0 and 49 (*p* < 0.001), Days 0 and 84 (*p* < 0.01), and Days 49 and 84 (*p* < 0.001) (Fig. 3A). Restricting the analysis to paired comparisons within the 35 children with growth measurements at all 3 timepoints, we confirmed the increased growth rate from Day 0 to Day 49 (*p* < 0.001) (Supplemental Fig. 3A).

To further confirm changes in malaria pathogenesis in RBCs from anemic children taking iron, we performed invasion assays to assess subjects' RBC susceptibility before and after iron supplementation in a subset of randomly selected subjects (*n* = 8). The mean SI values of these donors before iron supplementation (SI = 0.72; CI = 0.60–0.84) and post iron (SI = 1.58, CI = 1.17–1.99) were significantly different by student's *t*-test (*p* < 0.01) (Supplemental Fig. 3B).

### 3.6 The Population of Young RBCs Increases in Anemic Children Undergoing Iron Supplementation

To assess RBC population age structure, we evaluated levels of CD71-positive early reticulocytes in circulation at Days 0, 49, and 84 for a subset of anemic children undergoing iron supplementation. Relative percent of CD71-positive cells at Day 0 (mean = 129%, CI = 82–175%) was comparable to non-anemic controls (standardized as 100%), and increased at Day 49 (mean = 224%, CI = 166–286%) and Day 84 (mean = 180%, CI = 148–211%). Means were significantly different by one-way repeated measures ANOVA (*p* < 0.01), and Tukey's test showed significant difference between Days 0 and 49 only (*p* < 0.01) (Fig. 3B; Supplemental Fig. 3C).

**Supplemental Fig. 4:**
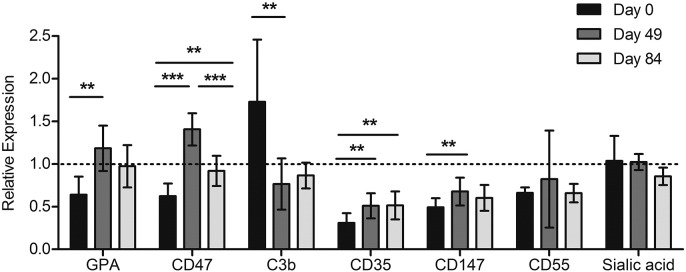
Surface markers of RBC age and integrity change in a pattern consistent with an increase in erythropoiesis in anemic children undergoing iron supplementation (12 mg daily). We measured GPA (an abundant sialoglycoprotein which contributes to RBC surface charge and is found at higher levels on younger RBCs (Beeson et al., 2016)), CD47 (an *anti*-phagocytic marker which influences RBC senescence and is found in lower levels in RBCs that have been in circulation longer or are less healthy (Lutz, 2004)), surface deposition of complement factor C3b (higher levels of which would correlate with increased RBC time in circulation, or less healthy RBC membranes (Gwamaka et al., 2012)), and levels of *P. falciparum* merozoite receptors (CD35, CD147, CD55, and sialic acid residues). Note that GPA is also a merozoite receptor, and CD35 and CD55 involved in the complement system have also been described as reflecting RBC age (more abundant on younger/healthier RBCs (Gwamaka et al., 2012)), as has sialic acid abundance (reduced on older RBCs) (Lutz, 2004). CD147, known as basigin, is the only known essential *P. falciparum* invasion receptor (Crosnier et al., 2011). Data represent relative expression based on anemic donor RBC MFI values (GPA, CD47, CD35, CD147, CD55, and sialic acid residues) or percent positive population values (C3b), compared to RBCs from a non-anemic donor not receiving iron supplementation (relative expression = 1.0). RBCs from the same 8 donors were examined over time. Error bars represent the 95% CIs. If indicated, one-way repeated measures ANOVA with post-hoc Tukey's test analysis indicates significant difference between expression levels (*p < 0.05, **p < 0.01, ***p < 0.001).

Further probing host factors which could increase parasite growth rates in RBCs from children undergoing iron supplementation, we assessed RBC surface markers from the same children over time (*n* = 8). We examined changes in surface expression of: glycophorin A (GPA), a sialoglycoprotein affecting RBC charge; CD47, an *anti*-phagocytic RBC marker; C3b deposition on RBC surfaces; CD35, complement receptor 1; CD55, a decay accelerating factor regulating complement on the cell surface; CD147; and sialic acid, all of which can reflect RBC age and overall membrane integrity and/or have been implicated in malaria merozoite invasion. We found significantly increased GPA, CD47, CD35 and CD147 levels and significantly decreased C3b deposition at Day 49 (*p* < 0.01 for all analyzing means between Day 0 and Day 49 by ANOVA and Tukey's test) (Supplemental Fig. 4). We were unable to detect differences in CD55 and sialic acid levels. Taken together, these surface marker findings support the idea that overall RBC population age and membrane physiology has shifted towards a younger, healthier RBC population following iron supplementation of anemic children.

## 4 Discussion

Use of *in vitro* growth assays as our primary outcome provided a rare opportunity to systematically examine the cellular determinants of parasite growth in anemic and iron-supplemented children. We demonstrate here that blood stage *in vitro P. falciparum* growth is decreased in RBCs from anemic children and this effect is reversed by iron supplementation.

Defining iron deficiency in children with ongoing infections or inflammation is difficult, and has confounded previous clinical studies trying to determine the protective effect of iron deficiency on malaria susceptibility. Here we show protection offered by anemia is substantial (~ 10% per standard deviation shift in hemoglobin), and RBCs from children with iron deficiency – no matter the definition criteria nor the presence of potential confounders such as inflammation – consistently reduce parasite growth compared to RBCs from non-anemic individuals. Additionally, the use of clinical parasite isolates from The Gambia confirms that this is not merely an artefact of laboratory strains. Notably, at the population level, anemia was estimated to confer at least four times as much protection against blood stage parasite growth than sickle-cell trait. Taken together, this data is evidence that anemia exhibits a profound natural influence on parasite growth beyond even the mostly commonly studied and referenced RBC polymorphisms which evolved due to malaria pressure.

Furthermore, we demonstrate parasite growth increases dramatically relative to baseline in RBCs taken from children during iron supplementation, transiently rising at Day 49 to exceed growth rates in non-anemic controls and remaining elevated at Day 84 relative to baseline. Iron deficient RBCs have a shorter circulation lifetime (90 *vs* 120 days, on average) and exhibit physiological differences such as microcytosis, decreased deformability, and increased oxidative membrane stress, among other effects – similar to changes in aged RBCs (Brandão et al., 2009). As parasites preferentially infect young RBCs and reticulocytes (Clark et al., 2014a, Lim et al., 2013), we assessed surface markers reflecting RBC age and integrity to provide a picture of the overall health of RBCs in anemic children undergoing iron treatment. Our data suggests that erythropoiesis increased in response to iron, creating a younger population of circulating RBCs. These younger RBCs are most prevalent at Day 49, which matches the largest shifts in malaria growth rates and supports our hypothesis that parasite growth transiently increases following iron supplementation due to *P. falciparum's* preference for young RBCs (Clark et al., 2014a). The study was constrained by the wide intervals between venous bleeds selected for the intervention. At Day 49, it is possible the main iron-induced erythropoietic surge already passed, in which case our data would underestimate the true extent of increased malaria risk.

We also examined merozoite invasion into RBCs from anemic and non-anemic individuals, as our previous work found invasion differences contributed significantly to reduced malaria pathogenesis in iron deficient RBCs (Clark et al., 2014a). We expanded our previous findings to show that RBCs from anemic African children were resistant to invasion with both laboratory and clinical *P. falciparum* strains and that iron supplementation increased invasion susceptibility. Our RBC surface marker data corroborating a shift towards younger, healthier RBCs corresponds with our hypothesis that changes in RBC population structure influence overall malaria risk.

The public health implications of our study are significant, shedding light on the overarching question of whether iron supplements cause harm. We acknowledge that *in vitro* parasite growth might not translate directly to malaria susceptibility. Yet there are no other viable alternatives for addressing this safety aspect regarding iron supplementation in malarious regions. While our system only examined the RBC impact of anemia on malaria growth, eliminating the impact of serum iron or immune cells, the fact that we still observe such profound growth effects highlights the protection afforded by anemia and the need for caution regarding iron supplementation. Furthermore, our results provide insight into why other clinical studies on this topic produce such variable results – given we find increased malaria susceptibility is transient, other studies may miss the window of enhanced susceptibility. We detect significant changes in parasite growth rates despite relatively small changes in hemoglobin levels, emphasizing the impact of iron and RBC population dynamics on *P. falciparum* pathogenesis. Our data clearly show that the safety of iron supplementation must be addressed, even if additional unknown mechanisms contribute to increased malaria susceptibility. We thus advocate temporary malaria prophylaxis should always accompany iron supplementation for anemic children in malaria endemic areas, though the period of enhanced susceptibility has not been accurately identified by this study. Finally, quantifying the sizeable contribution of anemia to population level protection against malaria, our research raises the question of whether consistently reduced hemoglobin and MCV values in people of African descent are genetic signatures of evolution under significant malaria pressure, much like the hemoglobinopathies.

The following are the supplementary data related to this article.

Supplementary material Image 1

## Role of the Funding Source

None of the funding sources had a role in study design, data collection or interpretation, writing of the manuscript, or the decision to submit for publication. The corresponding author had full access to all the data included in the study and assumed final responsibility for the decision to publish; all authors reviewed the report and agreed to submit for publication.

## Author Contributions

MMG, RW, AB, AMP, and CC designed the study and were involved in data analysis and interpretation, as well as writing. MMG, BD, ED, and DG participated in data collection. MA provided clinical *P. falciparum* isolates. JCP provided statistical support for data analysis. All authors reviewed and approved the final version.

## Declaration of Interests

We declare that we have no conflicts of interest.

## Figures and Tables

**Table 1:** Blood, inflammatory, and iron parameters of anemic donors whose RBCs were used for parasite growth assays before (Day 0), during (Day 49), and after (Day 84) iron supplementation. Tests were performed in MRCG Keneba laboratories using a Medonic M20 M GP and Cobas Integra 400 plus, or in the field using a HemoCue 301. Values in the Normal Range column are the normal or healthy range for each parameter for 6–24 month-olds as defined by standard guidelines. (Engorn, 2015). Numerical values reflect the mean value of all individuals and values in parentheses indicate standard deviation. Note that control non-anemic donors had an average hemoglobin of 14.13 g/dl (standard deviation 0.85).

Variable	Normal Range	Day 0 *n* = 158 Mean (SD)	Day 49 *n* = 91 Mean (SD)	Day 84 *n* = 87 Mean (SD)
White Blood Cell (× 10^9 per l)	6–17.0	12.11 (4.34)	12.35 (4.80)	12.22 (3.86)
Hemoglobin (g per dl)	11.0–13.5	9.88 (0.81)	10.68 (0.94)	10.78 (1.04)
Hematocrit (%)	33–39	28.88 (6.34)	28.57 (3.68)	29.67 (5.97)
Mean corpuscular volume (fl)	70–86	62.90 (7.66)	64.39 (6.40)	64.80 (6.15)
Mean corpuscular hemoglobin concentration (g per dl)	30–36	34.98 (1.47)	35.16 (1.32)	35.44 (1.18)
Red cell distribution width (%)	12–14	18.06 (2.51)	18.24 (2.38)	17.52 (2.17)
Platelet count (× 10^9 per l)	150–300	430.01 (200.10)	417.44 (172.28)	372.45 (155.27)
Iron total (μ mol per l)	9–21	4.99 (5.10)	9.24 (5.25)	14.97 (7.21)
Transferrin (g per l)	2–36	3.08 (0.62)	2.91 (0.52)	2.88 (0.56)
Transferrin saturation (%)	15–39	8.10 (8.76)	13.22 (6.73)	21.75 (11.04)
Ferritin (ng per ml)	12–140	16.55 (17.30)	28.81 (46.50)	22.78 (23.74)
Alpha 1 anti-glycoprotein (g per l)	< 1	1.29 (0.52)	1.27 (0.46)	1.29 (0.46)
C reactive protein (mg per dl)	0.8–3.1	6.30 (13.70)	5.19 (7.90)	4.56 (7.61)
Soluble transferrin receptor (nmol per l) (Vázquez-López et al., 2016)	1.26–1.23	8.83 (3.84)	8.21 (2.67)	7.36 (3.17)
Soluble transferrin receptor: log ferritin index	N/A	8.57 (18.24)	7.95 (9.10)	5.62 (7.39)
Hepcidin (ng per ml)	N/A	12.07 (13.73)	13.23 (12.76)	14.42 (12.37)

**Table 2:** Effect of host hemoglobin, iron status, and other hematological characteristics on *in vitro P. falciparum* growth in RBCs from anemic children (Hgb < 11 g/dl) at baseline. Growth rates (GR) were calculated relative to growth in healthy, non-anemic donors. Growth assays were performed in triplicate for each donor and the average value was used for linear regression modelling; multivariate analyses represent the estimated association for a given variable while controlling for potential confounders. Hgb genotype was evaluated solely based on AA *vs.* AS classification (too few individuals for statistical evaluation of SS genotypes) and G6PD status was evaluated solely based on normal *vs.* deficient classification. For continuous variables, the β_1_ value represents the %GR change (× 100) for every 1 unit increase in the primary variable. For categorical variables, the β_1_ value represents the %GR change (× 100) based on yes-no genotype. For example, for Hgb AS, the %GR change is − 18.3% relative to Hgb AA. Significant *p* values (< 0.05) are bolded. The standardized %GR change for Hgb and MCV is calculated based on the SD for the exposure variable of interest (see Table 1) multiplied by β_1_ (× 100%), to give the %GR change for every 1 SD change in the exposure variable; for Hgb genotype the standardized %GR change is simply β_1_ (× 100%).

Condition	β_1_ Value	Lower CI	Upper CI	*p* Value	Standardized % GR Change
*Bivariate analysis of measures affecting parasite growth*					
Hgb (g/dl)	0.107	0.039	0.174	**0.002**	8.6%
Hgb genotype (AA *vs* AS)	− 0.183	− 0.318	− 0.047	**0.009**	− 18.3%
MCV (fL)	0.014	0.007	0.021	**<** **0.001**	10.8%
G6PD status (normal *vs* deficient)	0.051	− 0.206	0.309	0.696	
Ferritin (ng/ml)	0.002	− 0.002	0.005	0.290	
Hepcidin (ng/ml)	0.004	0.000	0.008	0.074	
CRP (mg/dl)	− 0.002	− 0.006	0.002	0.360	
sTfR:log ferritin ratio	− 0.001	− 0.004	0.003	0.702	
Transferrin saturation (%)	0.431	− 0.307	1.169	0.255	
*Multivariate analysis of significant measures affecting parasite growth controlling for possible confounders*					
Hgb affects parasite growth controlling for Hgb genotype	0.103	0.036	0.170	**0.003**	8.3%
Hgb genotype affects parasite growth controlling for Hgb	− 0.179	− 0.312	− 0.047	**0.009**	− 17.9%
