# Supplementary material for: Anemia Offers Stronger Protection Than Sickle Cell Trait Against the Erythrocytic Stage of Falciparum Malaria and This Protection Is Reversed by Iron Supplementation
Source: eBioMedicine. 2016 Nov 9;14:123–30. doi: 10.1016/j.ebiom.2016.11.011 (PMC5161422; doi:10.1016/j.ebiom.2016.11.011)

Supplemental Information

Supplemental Methods

Micronutrient power: The iron supplementation product used in this trial is a micronutrient powder (MNP, MixMe WHO) produced by the DSM Company and distributed routinely by the United Nations Children's Fund and the World Food Program (contains 10 mg of iron). For our study purposes the iron concentration was altered to contain 12 mg iron/sachet (daily dose). The MNP contained Vitamin A (400 µg RE), Vitamin D (5 µg), Vitamin E (5 mg), Vitamin C (30 mg), Thiamine B1 (0.5 mg), Riboflavin B2 (0.5 mg), Niacin B3 (6 g), Pyridoxine B6 (0.5 mg), Cobalamine B12 (0.9 µg), Folate (150 g), Zinc (4.1 mg), Copper (0.56 mg), Selenium (17 µg), and Iodine (90 g).

Flow cytometry analysis: Flow cytometry was performed onsite at MRCG using a BD Accuri C6 flow cytometer. Channels and probes used included: SYBR Green I, FITC, and Alexa Fluor® 488 (488 nm excitation with a 530/30 nm bandpass emission filter, detector FL1); PE (488 nm excitation with a 585/40 nm bandpass emission filter, detector FL2); and CellTrace Far Red DDAO and Alexa Fluor® 647 (640 nm excitation with a 675/25 nm bandpass emission filter, detector FL4). Detector gain setting changes and compensation were not necessary with this configuration. Accuri C6 data was collected and analyzed with Accuri software (BD Accuri CSampler Analysis Software). Linear amplification of forward scatter was used to set event threshold in order to exclude cell debris, microparticles and doublets. For all experiments, samples were diluted to 0.001-0.002% hematocrit and ≥ 100,000 total events were collected.

RBC surface marker assessment: RBC surface protein levels were determined by staining with fluorescently tagged antibodies followed by flow cytometry analysis. The relative

expression fluorescence of each surface marker for each study sample was calculated using the MFI values (corrected for background), relative to MFI in the non-anemic donor. The exception was C3b for which the relative expression was reported as a percent of positively stained RBCs relative to the non-anemic donor.

Antibodies: The following antibodies were used: for CD35 (Mouse Anti-Human CD35 Clone E11 (BD) primary 1:2000; Alexa Fluor® 647 Rabbit Anti-Mouse IgG secondary 1:2000); for CD47 (Mouse Anti-Human CD47 Clone B6H12 primary 1:2000; Alexa Fluor® 647 Rabbit Anti-Mouse IgG secondary 1:500); for CD55 (Mouse Anti-Human CD55-PE conjugate Antibody NaM16-4D3 (Santa Cruz Biotechnologies) 1:10); for CD147 (Mouse Anti-Human CD147 Clone HIM6 (BD) primary 1:500; Alexa Fluor® 647 Rabbit Anti-Mouse IgG secondary 1:500); for Glycophorin A (GPA) (primary Rabbit Anti-Human CD235a/Glycophorin A (ThermoFisher Scientific/Pierce) 1:500 and secondary Alexa Fluor® 647 Goat Anti-Rabbit 1:2000); for sialic acid residues (Wheat Germ Agglutinin Alexa Fluor® 488 Conjugate (ThermoFisher Scientific/Molecular Probes) 1:2000); and for C3b deposition (primary Mouse Anti-Human Complement C3b Antibody 10C7 (ThermoFisher Scientific) 1:200; Alexa Fluor® 647 Rabbit Anti-Mouse IgG secondary 1:500).

Supplemental Tables, Figures, and Legends

Supplemental Table 1: Description of subjects. Hemoglobin genotyping was performed by electrophoresis. G6PD enzyme activity was performed by commercial kit. *A portion of subjects (% of total) were unable to be tested for G6PD status or hemoglobin genotype so are not included in the denominator for other % of total calculations.

**Description of study population at baseline**			
		All participants (n = 407)	Those to receive 12 mg iron daily (n = 135)
Age, months, mean (SD)		15.4 (4.4)	15.5 (4.4)
Sex (%)			
	Female	209 (51.2)	71 (52.6)
	Male	198 (48.5)	64 (47.4)
Z score, Wt for ht, mean (SD)		-0.91 (0.89)	-0.76 (0.84)
G6PD Status, n (% of those tested)			
	Normal	312 (93.7)	99 (93.3)
	Deficient	21 (6.3)	7 (6.6)
	Untested*	74 (18.2)	29 (21.3)
Hemoglobin genotype, n (%)			
	AA	326 (81.5)	110 (83.3)
	AS	66 (16.5)	19 (14.4)
	SS	6 (1.5)	2 (1.5)
	AC	1 (0.3)	0 (0.0)
	SC	1 (0.3)	1 (0.8)
	Untested*	7 (1.7)	3 (2.2)

Supplemental Table 2: **Blood, inflammatory, and iron parameters of study participants with reportable parasite growth rate data versus those without.** Numerical values reflect the mean value of all individuals of a particular category and time point, and values in parentheses are the SD.

**Variable**	**D0 w/ GR n = 158 Mean (SD)**	**D0 no GR n = 249 Mean (SD)**	**D49 w/ GR n = 91 Mean (SD)**	**D49 no GR n = 35 Mean (SD)**	**D84 w/ GR n = 87 Mean (SD)**	**D84 no GR n = 33 Mean (SD)**
White Blood Cell (x10^9 per l)	12.11 (4.34)	11.83 (3.82)	12.35 (4.80)	11.21 (2.69)	12.22 (3.86)	10.86 (2.54)
Red Blood Cell (x10^12 per l)	4.62 (1.00)	4.38 (0.63)	4.64 (0.62)	4.58 (0.42)	4.59 (0.85)	4.34 (0.33)
Hemoglobin (g per dl)	9.88 (0.81)	9.85 (0.82)	10.68 (0.94)	11.05 (1.21)	10.78 (1.04)	11.32 (1.04)
Hematocrit (%)	28.88 (6.34)	27.30 (3.90)	28.57 (3.68)	30.35 (3.93)	29.67 (5.97)	29.44 (3.00)
Mean Corpuscular Volume (fl)	62.90 (7.66)	62.72 (6.84)	64.39 (6.40)	66.27 (6.21)	64.80 (6.15)	67.84 (5.72)
Mean Corpuscular Hemoglobin (pg)	22.02 (2.92)	22.17 (2.84)	22.66 (2.56)	23.11 (2.43)	22.97 (2.33)	23.63 (2.20)
Mean Corpuscular Hemoglobin Concentration (g per dl)	34.98 (1.47)	35.29 (1.39)	35.16 (1.32)	34.88 (1.11)	35.44 (1.18)	34.82 (1.30)
Red Cell Distribution Width (%)	18.06 (2.51)	17.58 (2.68)	18.23 (2.38)	18.13 (2.46)	17.52 (2.17)	18.69 (2.21)
Mean Platelet Volume (fl)	7.62 (0.62)	7.70 (0.48)	7.76 (0.60)	7.85 (0.55)	7.90 (1.19)	7.62 (0.44)
Platelet Count (x10^9 per l)	430.00 (200.10)	459.21 (196.49)	417.44 (172.28)	386.64 (187.22)	372.45 (155.27)	370.35 (152.33)
Iron Total (µ mol per l)	4.99 (5.10)	6.18 (4.88)	9.24 (5.25)	9.53 (4.89)	14.97 (7.21)	17.23 (12.77)
Transferrin (g per l)	3.07 (0.62)	3.22 (0.66)	2.91 (1.52)	2.90 (0.48)	2.88 (0.56)	2.94 (0.54)
Transferrin Iron Binding Capacity (µ mol per l)	66.31 (11.50)	72.06 (16.95)	69.21 (12.40)	76.19 (11.88)	70.27 (13.62)	74.82 (11.45)
Transferrin Saturation (%)	8.10 (8.76)	9.04 (7.01)	13.22 (6.72)	12.60 (6.43)	21.75 (11.04)	23.12 (15.31)
Ferritin (ng per ml)	16.55 (17.30)	20.89 (28.33)	28.80 (4.50)	20.88 (13.58)	22.78 (23.74)	30.95 (27.07)
Alpha 1 Anti-glycoprotein (g per l)	1.29 (0.52)	1.24 (0.52)	1.27 (0.46)	1.02 (0.40)	1.29 (0.46)	1.04 (0.30)
C Reactive Protein (mg per dl)	6.30 (13.70)	6.27 (14.99)	5.19 (7.90)	4.87 (11.55)	4.56 (7.61)	3.15 (5.76)
Soluble Transferrin Receptor (nmol per l)	8.83 (3.84)	8.99 (3.84)	8.21 (2.67)	7.84 (2.47)	7.36 (3.17)	6.91 (2.13)
Soluble Transferrin Receptor: log Ferritin Index	8.57 (18.24)	7.09 (28.79)	7.95 (9.10)	4.43 (13.08)	5.62 (7.39)	4.51 (4.80)
Unsaturated Iron Binding Capacity (µ mol per l)	61.33 (13.17)	66.54 (17.17)	60.97 (11.91)	66.80 (11.75)	55.53 (15.23)	57.59 (15.47)
Hepcidin (ng per ml)	12.07 (13.73)	11.79 (11.61)	13.23 (12.78)	11.49 (12.89)	14.42 (12.37)	12.12 (10.95)

Supplemental Fig. 1: Parasite growth rates in RBCs from children categorized by different definitions of anemia at baseline. In analysis of parasite growth rates in RBCs from children at Day 0, we stratified participants (all anemic) using four different definitions to categorize the severity and type of iron deficiency in the presence or absence of inflammation: those with 1) hepcidin < 5.5 ng/ml (n = 82); 2) ferritin < 12 ng/ml (n = 69); 3) ferritin 12-30 ng/ml with CRP > 5 mg/ml (n = 17); 4) hemoglobin increase of >0.5 g/dl from baseline after 49 or 84 days of daily iron supplementation (n = 46); definitions 1-4 are not necessarily mutually exclusive. Of note, everyone in our population had a raised serum transferrin receptor (sTfR):log ferritin index >2 which is highly suggestive of iron deficiency. Growth rate values are presented relative to growth in RBCs from non-anemic donors. Each dot represents the mean result of triplicate growth assays from each donor and the error bars represent 95% CI. Mean growth rate results (with 95%CI) are: hepcidin < 5.5 ng/ml = 42.89% (37.11 - 48.67%); ferritin < 12 ng/ml = 43.34% (36.01 - 50.68%); ferritin 12-30 ng/ml with CRP > 5mg/ml = 49.08% (29.16 - 69.00%), ΔHgb > 0.5 g/dl = 44.04% (35.14 - 52.93%). There are no significant differences between the means.

Supplemental Fig. 1

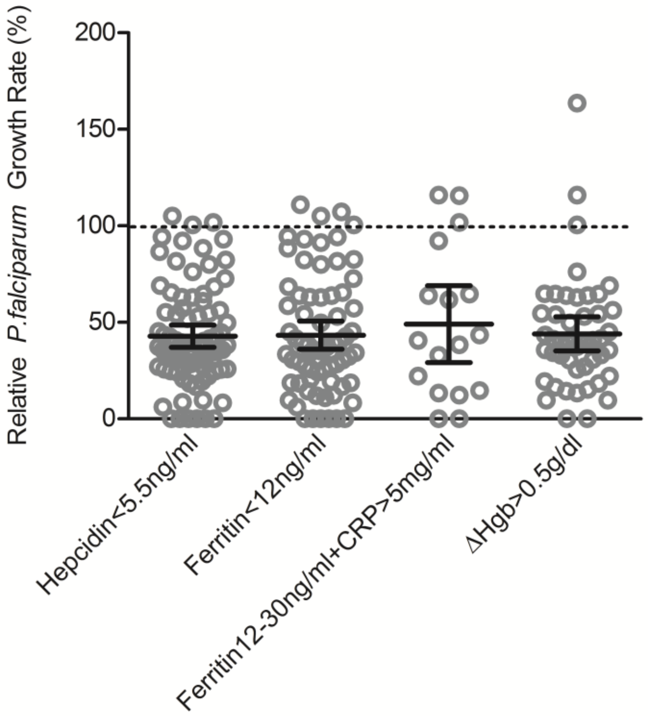

Supplemental Fig. 2: **Gating strategy to highlight adaptation of RBC barcoding assay to the field setting using basic two-color flow cytometry.**

a) RBCs from different blood donors are differentially labelled with CellTrace Far Red DDAO (1 µM (R3) or 0.1 µM (R4)) to distinguish between donor populations. Late stage purified parasites grown in unlabeled RBCs (R5) are seeded into the differentially labelled RBCs which have been combined in equal proportion.

b) M10 represents the 1 µM Far Red DDAO labelled RBCs from a non-anemic donor and M11 represents the 0.1 µM Far Red DDAO labelled RBCs from an anemic donor.

Gating cells on M11 (c) or M10 (d) allows for Sybr Green I DNA dye detection of parasite infected RBCs in the RBCs from anemic donors (M12, c) or from non-anemic donors (M13, d). Parasitemia in each cell population is compared to calculate the invasion SI. The percentages in the flow plots represent the percent of total cells within the indicated gate.

Supplemental Fig. 2

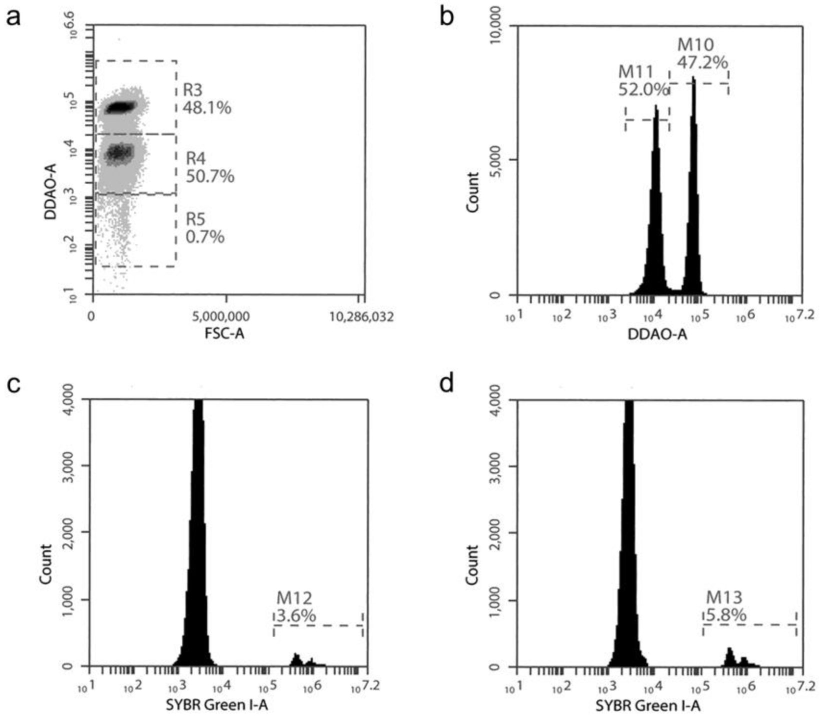

Supplemental Fig. 3: Changes in parasite growth, invasion, and reticulocytosis in RBCs from anemic children before and after daily iron supplementation.

a) Levels of parasite growth rates increase over time in anemic children undergoing iron supplementation, as depicted by line graph in order to highlight changes for each individual that had data available at all timepoints ($n = 35$ children with complete repeat growth measures at Day 0, 49, and 84, with 86% having increased growth rate at Day 49) One-way repeated measures ANOVA of growth rate values indicates the means are significantly different between Days ($p < 0.0001$); post-hoc analysis with Tukey's test indicates significant differences between Day 0 and Day 49 means ($p < 0.001$) and Day 49 and Day 84 means ($p < 0.001$), but no significance between Day 0 and Day 84 for those children with repeat measures.

b) Direct comparison of invasion into RBCs from non-anemic donors to RBCs from 8 anemic children either before or during 12 mg daily iron supplementation. Each experiment was performed in triplicate for each blood donor. The marker represents the SI point estimate and the bar represents the 95% CI. An SI of 1.0 indicates no difference in parasite invasion of the two RBC populations. Student's t-test indicates significant differences between pre- and post-iron SI values (**$p < 0.01$).

c) Line graph of CD71 repeated measures ($n = 31$ children with complete repeat CD71 measures at Day 0, 49, and 84). In 21 of these children, the relative percent CD71 positive cells increased from Day 0 to Day 49. See Fig. 3B for repeated measures ANOVA statistics.

Supplemental Fig. 3

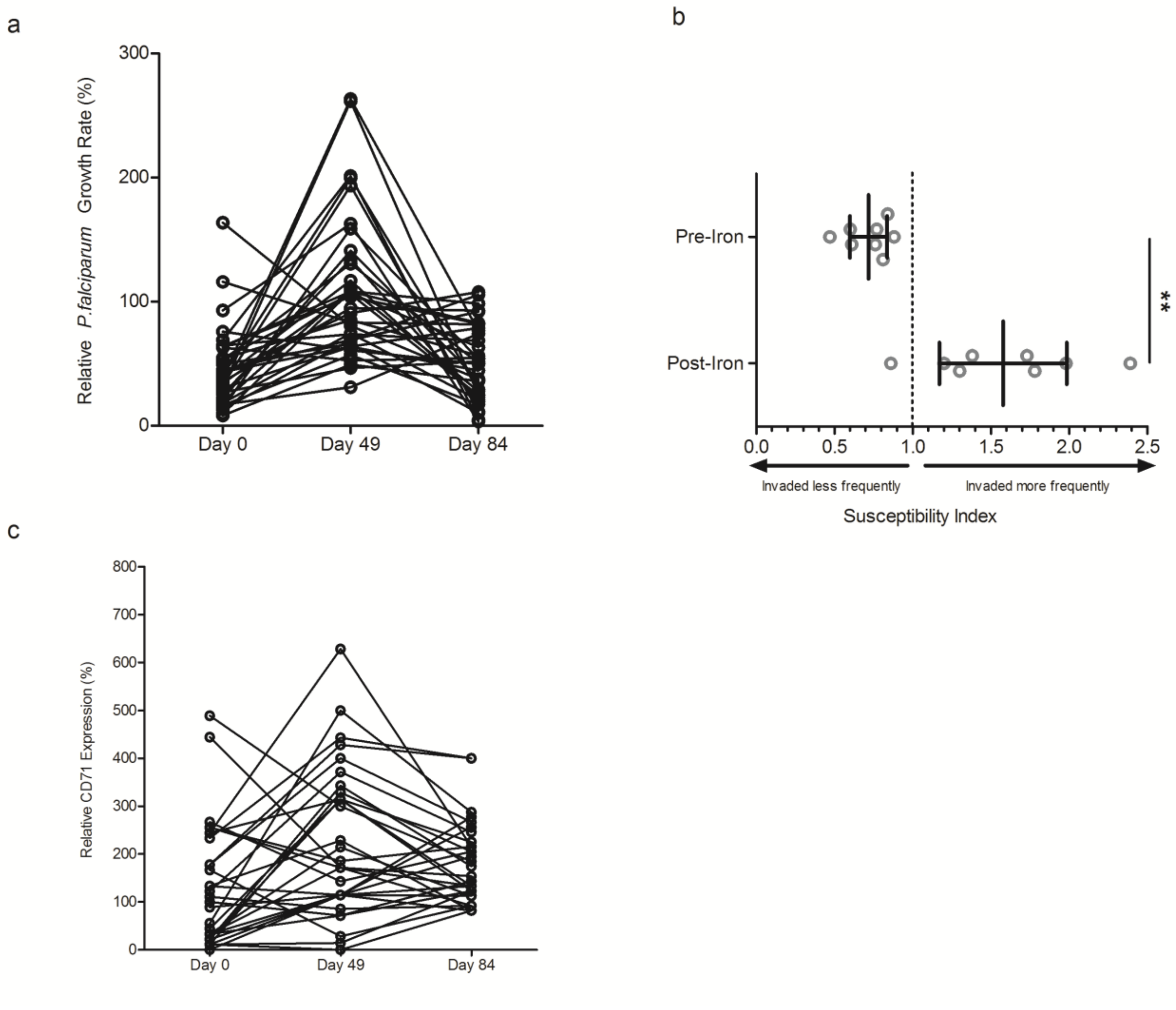

Supplemental Fig. 4: Surface markers of RBC age and integrity change in a pattern consistent with an increase in erythropoiesis in anemic children undergoing iron supplementation (12mg daily). We measured GPA (an abundant sialoglycoprotein which contributes to RBC surface charge and is found at higher levels on younger RBCs (Beeson et al., 2016)), CD47 (an anti-phagocytic marker which influences RBC senescence and is found in lower levels in RBCs that have been in circulation longer or are less healthy (Lutz, 2004)), surface deposition of complement factor C3b (higher levels of which would correlate with increased RBC time in circulation, or less healthy RBC membranes (Gwamaka et al., 2011)), and levels of *P.falciparum* merozoite receptors (CD35, CD147, CD55, and sialic acid residues). Note that GPA is also a merozoite receptor, and CD35 and CD55 involved in the complement system have also been described as reflecting RBC age (more abundant on younger/healthier RBCs (Gwamaka et al., 2011)), as has sialic acid abundance (reduced on older RBCs) (Lutz, 2004). CD147, known as basigin, is the only known essential *P.falciparum* invasion receptor (Crosnier et al., 2011). Data represent relative expression based on anemic donor RBC MFI values (GPA, CD47, CD35, CD147, CD55, and sialic acid residues) or percent positive population values (C3b), compared to RBCs from a non-anemic donor not receiving iron supplementation (relative expression = 1.0). RBCs from the same 8 donors were examined over time. Error bars represent the 95% CIs. If indicated, one-way repeated measures ANOVA with post-hoc Tukey's test analysis indicates significant difference between expression levels (*$p < 0.05$, **$p < 0.01$, ***$p < 0.001$).

Supplemental Fig. 4

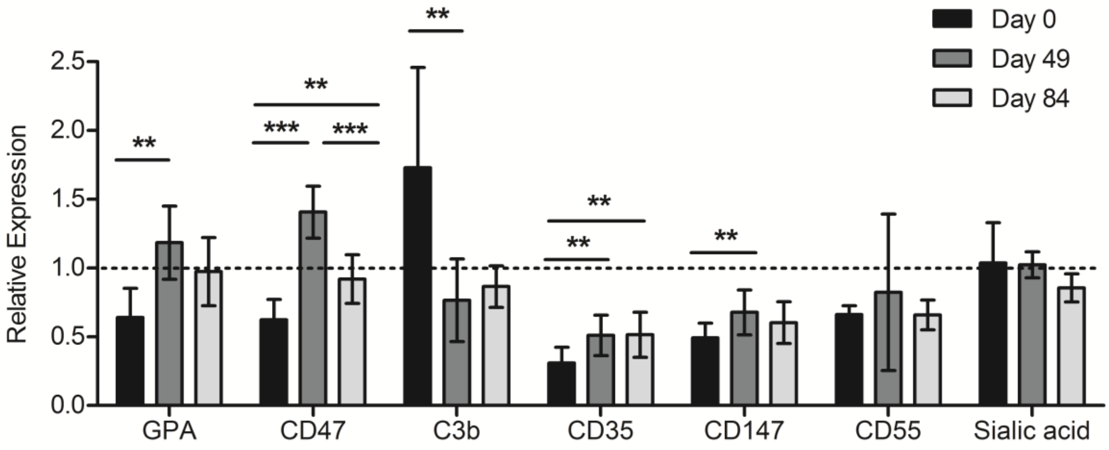

Supplement: Supplementary file 1 — Supplementary material [file mmc1.pdf]
